# The efficacy of the traditional Chinese medicine Jia Wei Niu Bang Zi granule combined with methotrexate in treating active rheumatoid arthritis: A multicenter, randomized, double-blinded controlled clinical trial

**DOI:** 10.1097/MD.0000000000014424

**Published:** 2019-02-08

**Authors:** Yi-Ru Wang, Li Liu, Xiao-Yun Wang, Qiong Wang, Min Yao, Xue-jun Cui, Jian-Chun Mao, Jian Liu, Xiao-Hua Gu, Yong-Li Chen, Xiang Yu, Qi Shi, Qian-Qian Liang, Yong-Jun Wang

**Affiliations:** a Longhua Hospital, Shanghai University of Traditional Chinese Medicine; b Institute of Spine, Shanghai University of Traditional Chinese Medicine; c Key Laboratory of Theory and Therapy of Muscles and Bones, Ministry of Education (Shanghai University of Traditional Chinese Medicine); d Department of Rheumatology, Longhua Hospital, Shanghai University of Traditional Chinese Medicine; e Department of Rheumatology, The First Affiliated Hospital of Anhui University of Chinese Medicine, Hefei; f Department of Orthopaedics, Shanghai Seventh People's Hospital, Shanghai; g Department of Rheumatology, The Bengbu Hospital of Chinese Medicine, Bengbu; h Rehabilitation Medicine College, Shanghai University of Traditional Chinese Medicine, Shanghai, China.

**Keywords:** active rheumatoid arthritis, Jia Wei Niu Bang Zi granule, methotrexate, randomized controlled trial, traditional Chinese medicine

## Abstract

**Background:**

Rheumatoid arthritis (RA) is a chronic systemic and autoimmune inflammatory disease ending with the destruction of joints. Current therapies can relieve RA symptoms, but some also bring severe adverse events. Therefore, an effective and safe therapeutic strategy remains to be created to benefit patients with RA by large. Jia Wei Niu Bang Zi granule (NBZG) consisting of RA-fighting Chinese herbals has been used in Longhua Hospital in the last several decades. NBZG has potential therapeutic effect on RA, which should be evaluated by larger sample clinical trial.

**Methods:**

A multicenter, randomized, double-blind, placebo-controlled clinical trials will be conducted to determine the efficiency of NBZG in pain relief and joint protection. A total of 120 patients with active RA will be enrolled, and treated with NBZG or placebo for 12 weeks. The primary outcome measurements include rate of American College of Rheumatology (ACR) 50 at 12 weeks’ treatment. The 2nd outcome measurements include rate change of ACR20, ACR70, the disease activity score (DAS) 28, 36-item Short-Form Health Survey Questionnaire, Health Assessment Questionnaire - Disability Index, score changes of Patient Assessment of Arthritis Pain, Patient Global Assessment of Arthritis, and the Athens insomnia scale at the same time points.

**Discussion:**

Although NBZG has shown efficacy in treating RA in Longhua Hospital for decades, the universality of this efficacy needs evaluated. The results of this trial will provide a convincing evidence about NBZG's efficacy in treating active RA in a large population.

**Trial registration:**

ClinicalTrials.gov ID: NCT03173040 (registered on May 30, 2017)

## 1 Introduction

Rheumatoid arthritis (RA) is a chronic inflammatory joint disease, which usually causes progressive joint destruction, premature unemployment, physical disability, and short-life expectancy.^[1–3]^ Treatment procedures include measuring disease activity with comprehensive indexes, applying treatment-to-target strategy with conventional, biologic (or nonbiologic) disease-modifying antirheumatic drugs. When the disease symptoms remit and stabilize (e.g., showing a low activity), the dose is reduced. With this treatment, most patients with RA show a favorable outcome, but many still do not respond appropriately. Therefore, novel treatments are urgently demanded.

For centuries, herbal medicine has been used as a complementary and alternative medicine for western medicine in China.^[4,5]^ According to the theory of traditional Chinese medicine (TCM), RA can be defined as a Bi syndrome. To deal with RA, Jia Wei Niu Bang Zi decoction (JWNBZ) was established by a group of TCM doctors in China, using fructus arctii as the principal element, and *Bombyx batryticatus*, pseudo-ginseng radix angelicae tuhuo, large Leaf Gentian and *Pinellia ternata* as adjuvant components. JWNBZ could reduce synovial inflammation, bone erosion, and osteoclast number at ankle joints in TNF-Tg mice without triggering any side effect,^[6]^ suggesting the therapeutic potential of JWNBZ against human RA. But this efficacy should be verified with more researches.

We will conduct a well-designed, randomized, double-blind, placebo-controlled trial to estimate the effectiveness and safety of JWNBZ on RA. The outcomes of this clinical trial can evidence the value of JWNBZ to weak disease activity and protect joints from deformity. To guarantee the quality of medicine during the whole trial, we will use JWNBZ granule (NBZG), raw extraction of NBZG decoction, to replace medicinal broth.

## 2 Materials and methods

### 2.1 Study design

This study is a multicenter, randomized, double-blinded, placebo-controlled clinical trial with 3 parallel arms. The aim of the study is to evaluate the effectiveness and safety of NBZG combined with methotrexate (MTX) in managing active RA. MTX is the 1st-line medicine on the treatment of RA. In total, we will recruit 120 patients with active RA (30 patients per arm) from four hospitals in China: Longhua Hospital affiliated to Shanghai University of Traditional Chinese Medicine, The First Affiliated Hospital of Anhui University of Chinese Medicine, Shanghai Seventh People's Hospital and Bengbu Hospital of Chinese Medicine. All participants will be randomized into NBZG group or placebo group equal in patient number. Patients in NBZG group will receive both NBZG (4 g, twice a day) and MTX (5 mg per week), while patients in placebo group will receive NBZG placebo (4 g, twice a day) and MTX (5 mg per week) for continuous 12 weeks. The Standard Protocol Items: Recommendation for Interventional Trials (SPIRIT) 2013 Checklist.

### 2.2 Ethical issues

The trial will be conducted in accordance with the Declaration of Helsinki and Ethical Guidelines for Clinical Research, and the trial protocol has been approved by the Research Ethical Committee of Longhua Hospital, affiliated to Shanghai University of Traditional Chinese Medicine, Shanghai, China (approval number: 2017LCSY011), The First Affiliated Hospital of Anhui University of Chinese Medicine (approval number: 2018AHKYBF01), Shanghai Seventh People's Hospital (approval number: 2018-IRBQY-007), and Bengbu Hospital of Chinese Medicine (Approval Number: 2018-BBZYY006) (additional files 1–4). And the protocol has been registered on Clinical Trials (ClinicalTrials.gov, ID: NCT03173040).

### 2.3 Study participants

Participants will be recruited from 4 hospitals (Longhua Hospital Affiliated to Shanghai University of Traditional Chinese Medicine, The First Affiliated Hospital of Anhui University of Chinese Medicine, Shanghai Seventh People's Hospital, and Bengbu Hospital of Chinese Medicine). Recruitment poster and website advertisements will be publicized. The planned recruitment period is 12 months.

### 2.4 Sample size calculation

We calculated the sample size according to our primary study. We conducted a preliminary experiment from November 2016 to July 2017 into the efficacy of the NBZP combined with MTX vs MTX alone, and we found that patients taking the NBZP combined with MTX achieved 84.8% American College of Rheumatology (ACR) 50, whereas patients in the MTX group achieved 55.5% ACR50 response. The formula to calculate the rate in completely random design is: 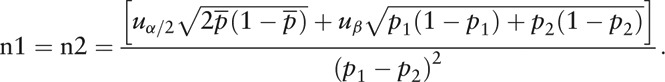


n1 and n2 are the patient numbers in NBZG group and placebo group; 
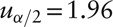
 when type 1 error is 0.05, *u*_*β*_ = 1.282 when type II error is 0.1 in 2-sided tests. 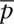 is the mean of *p*_1_ and *p*_2_.^[7]^ Approximately 120 participants are needed to achieve a 90% power and a (2-sided) 5% significance level in detecting the therapeutic differences between 2 groups. Thus, the final sample size has been set as 120 patients (60 per group), considering 20% dropout rate.

### 2.5 Criteria

#### 2.5.1 Inclusion criteria

Participants meeting the following criteria will be included.

- Having an age of ≥18 years.
- Meeting ACR/European League Against Rheumatism criteria for RA, 2010.^[8]^
- Showing symptoms within 12 months before enrollment.
- Showing high disease activity at enrollment (disease activity score [DAS] 28 score > 3.2),^[9]^ no prior exposure to oral glucocorticoids (>10 mg/d) or biologic agents.
- Being able to understand or sign an informed consent form.

#### 2.5.2 Exclusion criteria

- Presenting autoimmune or inflammatory disease at joints such as adjuvant arthritis, lupus arthritis, osteoarthritis, gouty arthritis, ankylosing spondylitis, infectious arthritis, psoriasis arthritis, enteropathy arthritis, Sjogren syndrome, scleroderma, and cancer.
- Showing abnormal liver and kidney function.
- Being pregnant or having a plan of pregnancy (breast feeding women).
- Presenting severe chronic or acute disease that may drop the patients out.
- Being addictive to alcohol.

#### 2.5.3 Interventions

In NBZG group, patients will be instructed to dissolve NBZG (4 g) in 200 mL hot water and take the solution orally twice a day for 12 weeks, while patients in the placebo group will take NBZG placebo in the same way. Besides, patients in the 2 groups will be given MTX (5 mg per week), and glucocorticoid (<5 mg a day) if necessary, but not any other drugs, especially herbs.

The NBZG will be manufactured, packaged, and labeled by PuraPharm Corporation in Nanning. The crude herbs, including fructus arctii (Niu Bang Zi, 12.1 kg), *B batryticatus* (Jiang Can, 12.1 kg), *Panax notoginseng* (San Qi, 12.1 kg), *Angelica biserrata* (Du Huo, 12.1 kg), Radix Gentianae Macrophyllae (Qin Jiao, 7.9 kg), and Rhizoma Pinelliae (Ban Xia, 12.1 kg), will be supplied in 1 batch from Longhua Hospital and stored in a specialized cool and dry place. NBZG is made from the following ingredients: Fructus Arctii (chaoniubangzi, 9 g), Bombyx Batryticatus (Jiang Can, 9 g), Notoginseng Radix Et Rhizoma (San Qi, 9 g), Angelicae Pubescentis Radix (Du Huo, 9 g), Gentianae Macrop Hyllae Radix (Qin Jiao, 6 g), and Rhizoma Pinelliae (Ban Xia, 9 g). The processes involved are as follows:

1. Extraction: All the herbs mentioned above are put into a water-filled container for a 1-hour soak. And then the mixture is boiled at 100°C for 1 hour. Herbal dregs filtered out, and herbal filtrate collected.
2. Concentration: Herbal filtrate is concentrated.
3. Granule: Concentrate is mixed with dextrin. The mixture is spray dried to produce dry paste. Then the dry past mixed with magnesium stearate is made into powders, smashed, and screened through a mesh size of 80. Finally, the NBZGs are bagged (4 g per bag). NBZG placebo contains 10% NBZG and 90% bitterant, lactose edible essence, pigment (such as lemon yellow, caramel pigment, and sunset yellow) and starch, and exhibit shape, smell, color, and taste similar to those of NBZGs. For the control group, placebo granules are also made from dextrin (90%) and NBZG (10%) to achieve the same color, smell, taste, and texture as NBZG.

Participants can orally take glucocorticoid <5 mg a day if necessary. At baseline (Visit 1), patients begin to take NBZG or placebo NBZG and laboratory tests. At weeks 4, 8, and 12, patients will visit again (Visits 2 and 4) and perform laboratory tests. Then at week 16, the final visit (Visit 5) will be taken through a phone call. Every patient should visit within 3 days after the arranged time point.

We will call the patients to take laboratory tests to record in a notebook the medicine they have used. When serious adverse effects occur, we will provide an appropriate treatment to the patient immediately, record the adverse effect, and stop medicine.

### 2.6 Randomization and allocation

The random number list will be generated through Statistical Product and Service Solutions 22.0 (IBM SPSS) in a 1:1 ratio by a trained statistician. A stratified random method will be used to balance the patients within hospitals and the stratification factor will be the 4 study centers. The list contains 2 corresponding columns, 1 is the sequence number of inclusion, the other 1 is the treatment or placebo group. This statistician will contact the pharmaceutical factory and give them this randomization list. The factory than will stick the sequence number label on boxes of NBZG or placebo as the list. The statistician and pharmaceutical factory will store the randomization list to maintain concealment. And the other investigators will not know the corresponding relations between sequence numbers and different groups until the trials completed.

Medicine (NBZG or the placebo) with labels will be stored in a locked and appropriate room. A trained administrator will charge the medicine and the key. And this administrator also will charge the assignment of participants to interventions.

When a participant is recruited in, the investigator will make a central telephone call and give him/her the medicine (NBZG or the placebo) from the administrator according to the central call.

### 2.7 Blinding

The stuff in the pharmaceutical factory only provides drugs and random number list, and never participates in the trial. The investigators, clinicians, nurses, outcome evaluators, statisticians, and participants have no idea about the group assignment information until the entire trial is completed.

When an adverse event is claimed, we will record it and provide an appropriate treatment. Clinicians will provide emergency services in case of serious adverse events, and report them to the Institutional Review Board within 24 hours. Then the blinding will be broken by the main trial investigator, leader of Longhua Hospital ethic committee and the statistician to provide participant information of intervention, NBZG or NBZG placebo.

### 2.8 Outcomes

#### 2.8.1 Primary outcomes

The ACR50 rate after 12 weeks’ treatment was compared to that at baseline in the 2 groups. The ACR50 is a scale to measure change in RA symptoms,^[10]^ which have been used for testing the effectiveness of clinical interventions in large-sample groups,^[11,12]^ ACR 50 is met when the number of tender joints reduces by ≥50%, the number of swollen joints by ≥50%, and at least 3 of the following 5 indexes improved by ≥50%:

- Patient's arthritis pain using a visual analog scale (VAS) of 0 to 100 mm
- Patient's disease activity using VAS (0–10)
- Patient's physical function and disability using Health Assessment Questionnaire - Disability Index (HAQ-DI)
- Acute-phase reactant value such as erythrocyte sedimentation rate (ESR) or C-reactive protein level

Similarly, ACR20 is met when ≥20% is achieved in the reduction of tender joint number, swollen joint number, and the rise of the score of at least 3 of another 5 indexes.

Although compared to ACR50 and ACR70, ACR20 has been accepted as a benchmark in RA clinical trials for its capacity to distinguish active treatment from placebo control,^[13,14]^ ACR50 is still a preferred cutoff than ACR70 to evaluate RA.^[11]^ Here, we choose ACR50 to measure the primary outcome.^[11,15]^

### 2.9 Secondary outcomes

The secondary outcome parameters include the DAS28, rate of ACR20/70, HAQ-DI, 36-item Short-Form Health Survey Questionnaire (SF-36), Patient Assessment of Arthritis Pain, Patient Global Assessment of Arthritis, and Athens insomnia sleep scale.

#### 2.9.1 DAS28: from baseline to weeks 4, 8, 12, and 16

The DAS28, a widely used indicator to evaluate RA disease activity after treatment, was published in 1993 by van der Heijde et al.^[16]^ DAS includes the following 4 measurements^[17]^: the counts of swollen and painful joints, the ESR, and a measurement for general health (GH). The formula of DAS28 is 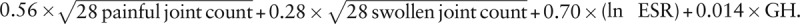 The 28 joints include 10 proximal interphalangeal joints and 10 proximal interphalangeal joints of hands and wrists, elbows, knees, and shoulders bilaterally. GH is patient GH VAS (0–10 mm). Using this formula, DAS28 <2.6 signifies remission.

#### 2.9.2 HAQ-DI (from baseline to weeks 4, 8, 12, and 16)

The HAQ-DI, a modification of ACR20/50/70, is efficient to assess functional status of the patient attacked by chronic illness,^[12]^ like RA.^[18]^

There are 20 questions that describe 8 movements: dress, rise, eat, walk, hygiene, reach, grip, and usual activities. Each question is followed by 4 choices: without any difficulty (0 point); with some difficulty (1 point); with much difficulty (2 points); and unable to do (3 points). HAQ-DI score is the average of 8 sections’ scores ranging between 0 and 3, higher score representing more severe disability. Generally, HAQ-DI score of 0 to 1 indicates mild to moderate difficulty; 1 to 2 indicates moderate to severe disability; 2 to 3 indicates severe to extremely severe disability.^[19]^

#### 2.9.3 Patient assessment of arthritis pain and patient global assessment of arthritis (from baseline to weeks 4, 8, 12, and 16)

Patient assessment of arthritis pain and patient global assessment of arthritis are 2 subscales of ACR 20/50/70 and use a VAS of 0 to 100 mm.^[12]^

#### 2.9.4 Athens insomnia scale (from baseline to weeks 4, 8, 12, and 16)

Most patients with RA report poor sleep,^[20]^ which may be induced by pain, fatigue, depression,^[21]^ and inflammation.^[22]^ Athens insomnia scale (AIS), a useful tool in sleep research and clinical practice, consists of 8 items: sleep induction, awakenings during the night, final awakening, total sleep duration, sleep quality during the night, emotion during the day time, functioning capacity, and sleepiness during the day time.^[23]^ Each item gives 0 to 3 points, with 0 representing no problem at all and 3 representing very serious condition. Thus, the total maximum score is 24 which means the most severe degree of insomnia. The lower score indicates the better sleep quality.^[24]^

#### 2.9.5 SF-36 (from baseline to weeks 4, 8, 12, and 16)

The SF-36 is a tool for assessing physical status of patients with RA.^[25–27]^

SF-36 consists of 36 items in 8 types, including physical function, role physical, bodily pain, and global health, vitality, social function, role emotional, and mental health.^[28]^

### 2.10 Safety assessments

To assure the safety of participants, the data collection process will be supervised by the project director. The patients’ blood routine, urine routine, feces routine, kidney, and liver function will be tested on schedule. At each visit, patients will be asked about the adverse effects during the study period. To protect their privacy, all participants will be visited in a closed room. Participants can raise questions and withdraw at any time.

### 2.11 Participant timeline

**Figure 1:**
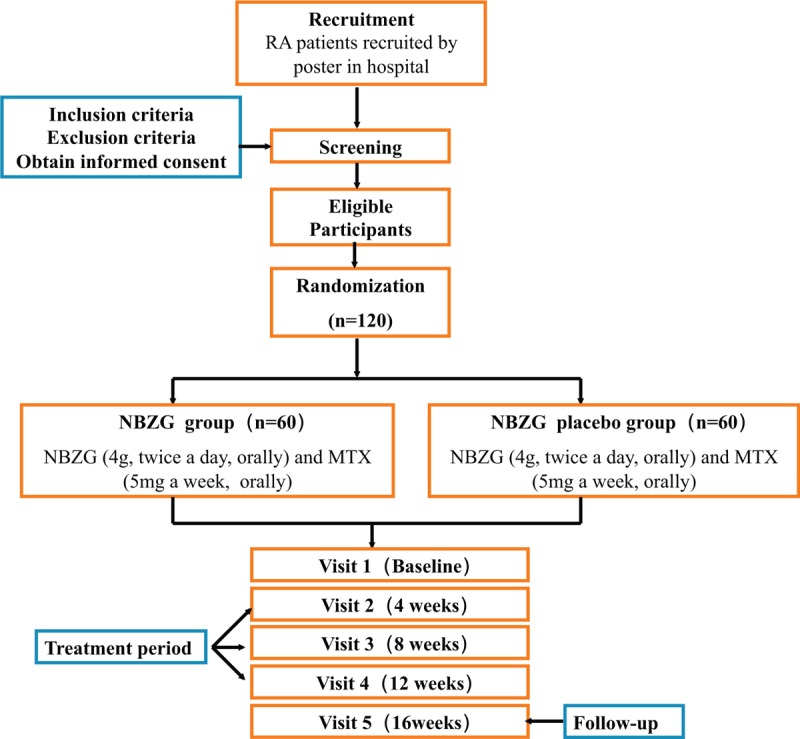
Project overview. RA = rheumatoid arthritis, NBZG = Jia Wei Niu Bang Zi granule.

Recruitment will start in February 2019 and end in February 2020. The last visit will be finished before March 31, 2021. The recruitment process is shown in Figure 1, and the schedule is shown in Table 1 table 1.

**Table 1:**
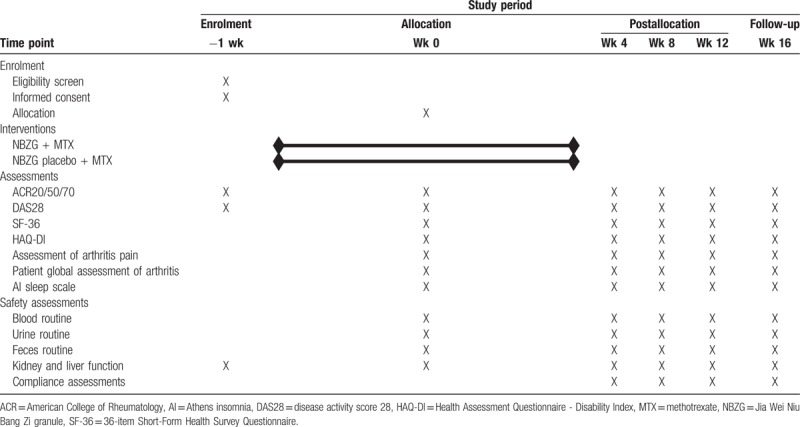
Schedule of enrollment and assessments.

### 2.12 Data collection and monitoring

During this 16-week trial, all participants will be treated with NBZG or NBZG placebo for 12 weeks follow-up for 4 weeks. Five rounds of disease activity assessments (the baseline, week 4, week 8, week 12, and week 16) and 5 rounds of safety assessments (the baseline, week 4, week 8, week 12, and week 16) will be recorded in Epidata (Version 3.1) by 2 statisticians independently. Disagreement will be solved by discussion and a third statistician.

Longhua Hospital Affiliated to Shanghai University of Traditional Chinese Medicine (http://www.longhua.net/ywsy/gzlc/287.jhtml) control the quality.

### 2.13 Quality control

Training will be carried out before the trial to let the involved physicians, nurses, and evaluators from the multicenter fully apprehend the process of the entire trial. When the trial starts, several investigators will be sent to 4 centers every month to assure whether: each center recruits enough participants in the plan; all participants meet the inclusion criteria and do not meet the exclusion criteria; all the participants fully follow the clinical trial process, and the case report form (CRF) has been completed. At the same time, we will call every participant to check the CRF and put its information into the e-database of CRF monthly. Epidata (version 3.0) procedure will be used to restrict data values. Two investigators will enter and compare the data independently to rule out the difference.

### 2.14 Statistical analysis

All data analyses will be performed using Statistical Packages of Social Sciences (SPSS) software (version 22.0). Continuous data are presented as mean ± standard deviation (SD) and categorical data as frequencies. Continuous variables following the normal distribution will be analyzed by Student *t* test; otherwise, nonparametric tests will be used to compare group differences. Statistical test is 2-sided and *P*-value <.05 will be defined as statistically significant. We will adopt intention-to-treat approach and last-observation-carried-forward method will be applied in the missing values.

## 3 Discussion

Fast-pace bioscience has dramatically improved the treatment of RA. But to benefit patients as many, these treatments wait to be updated. JWNBZ consists of fructus arctii, *B batryticatus*, pseudo-ginseng radix angelicae tuhuo, large-leaf Gentian, and *P ternata*. Our previous study proved that JWNBZ could ameliorate synovial inflammation and joint damage in TNF-Tg mice without any side effects,^[6]^ suggesting the potential therapeutic effect of NBZG on RA that should be evaluated by large-sample trial.

We retrieved CINAHL, Embase, Wan Fang Data, the Cochrane Library, PubMed, ISI web of knowledge, Vip Journal Integration Platform, Chinese BioMedical databases (Sinomed), CNKI (including China Doctor/Master Dissertation Full Text Database and China Proceedings Conference Full Text Database), and clinical trials up to April 2018, finding no evidence about the effectiveness and safety of NBZG in treating active RA. Therefore, our study will be the 1st multicenter, randomized, double-blind, placebo-controlled clinical trial that investigates the efficacy and safety of NBZG combined with MTX in treating active RA.

To assure the research reliability, we plan to conduct a 5-center, randomized, controlled clinical trial. In addition, DAS28 and ACR50 will be applied to assess the injury and disease activity of the RA joints. Furthermore, we will evaluate the patients’ quality of life using AIS, HAQ-DI, and SF-36. The safety of NBZG will be evaluated according to patients’ feeling and laboratory tests, such as feces routine, blood routine, urine routine, kidney, and liver function. Ethical evaluation will be strictly made and patients’ compliance closely observed, because there is inadequate evidence about the effectiveness of NBZG on RA (MTX is a 1st-line drug in Disease Modifying Anti Rheumatic Drugs which can achieve DAS28 in 53.8% of the patients).

This research will answer whether NBZG combined with MTX is an effective strategy to treat active RA. Its positive results will offer patients with RA and doctors a novel treatment choice. Furthermore, the clinical trial can provide evidence about the effect of NBZG on life quality, drug safety, joint function, and symptom severity. Its findings can also be written into new guidelines about RA treatment. Recruitment will start in February 2019 and is expected to finish in February 2020.

## Author contributions

LL, WYR, and WXY are co-first authors of this manuscript, contributing equally to the design, conduct the trials, and draft the manuscript. All authors participated in the design of the study and performed the trial. LQQ, SQ, and WYJ supervised and coordinated the clinical trial. LL, MJC, WXY, GXH, LJ, YX, and CYL are responsible for recruiting the participants. WQ, YM, and CXJ are participated in statistical design. All authors read and approved the final manuscript. SQ, LQQ, and WYJ conceived of the study and revised the manuscript critically for important intellectual content.

**Funding acquisition:** Qi Shi, Qian-Qian Liang, Yong-Jun Wang.

**Investigation:** Yiru Wang, Jian-Chun Mao, Jian Liu, Xiao-Hua Gu, Yong-Li Chen, Xiang Yu.

**Methodology:** Li Liu, Xiao-Yun Wang, Qiong Wang, Min Yao, Xue-jun Cui.

**Project administration:** Qian-Qian Liang.

**Supervision:** Qi Shi, Qian-Qian Liang, Yong-Jun Wang.

**Writing – original draft:** Yiru Wang, Li Liu.

**Writing – review & editing:** Yiru Wang.
